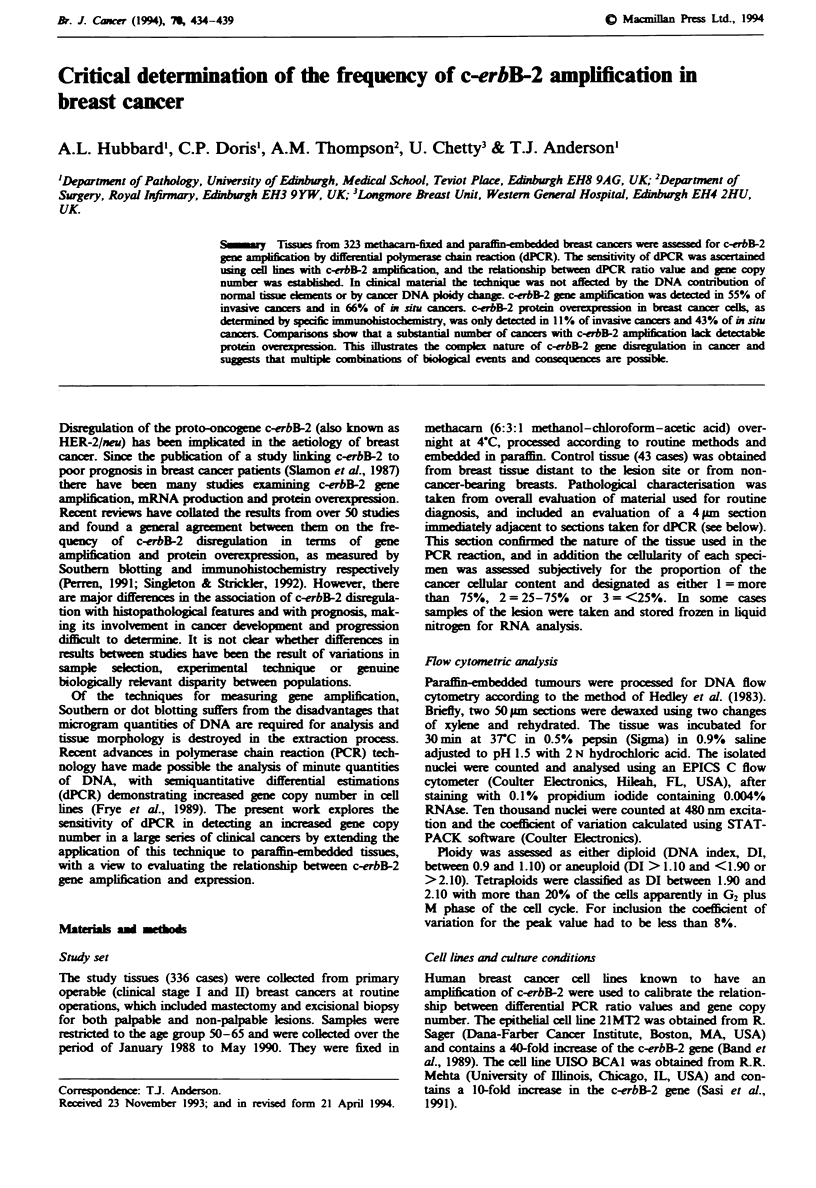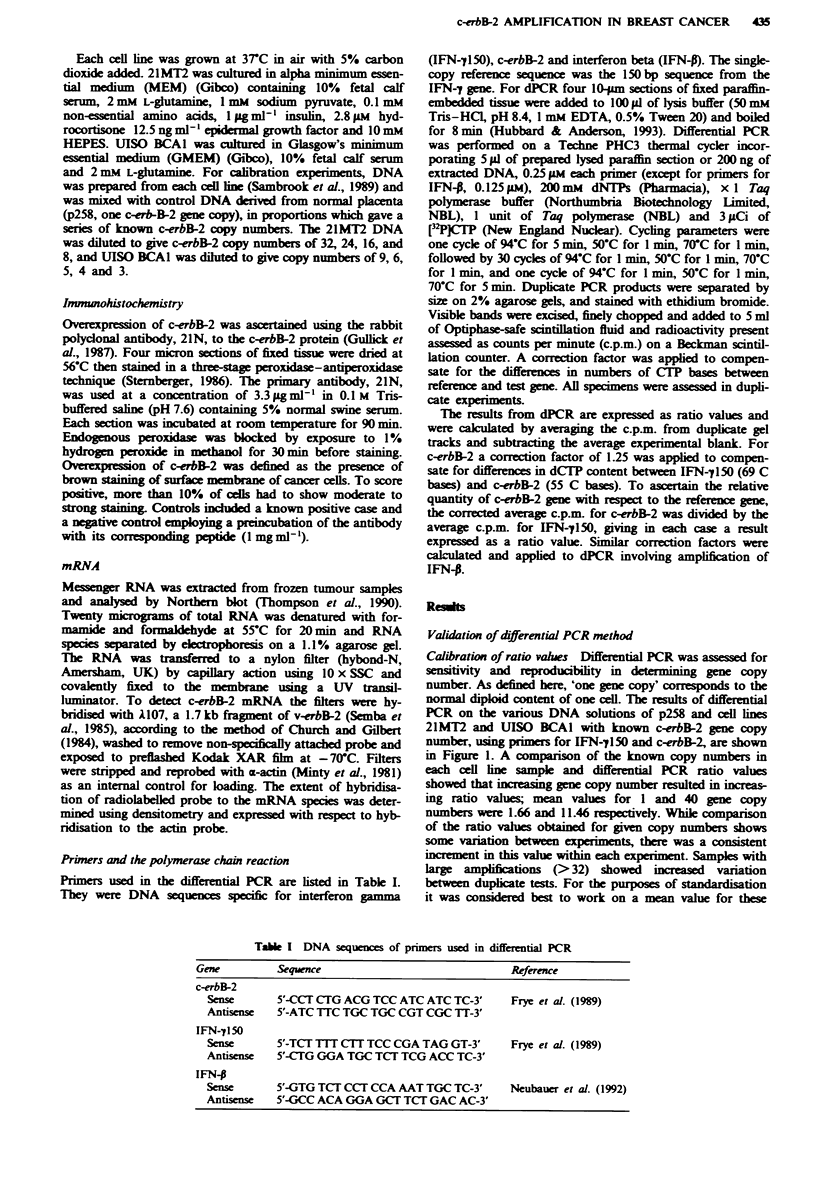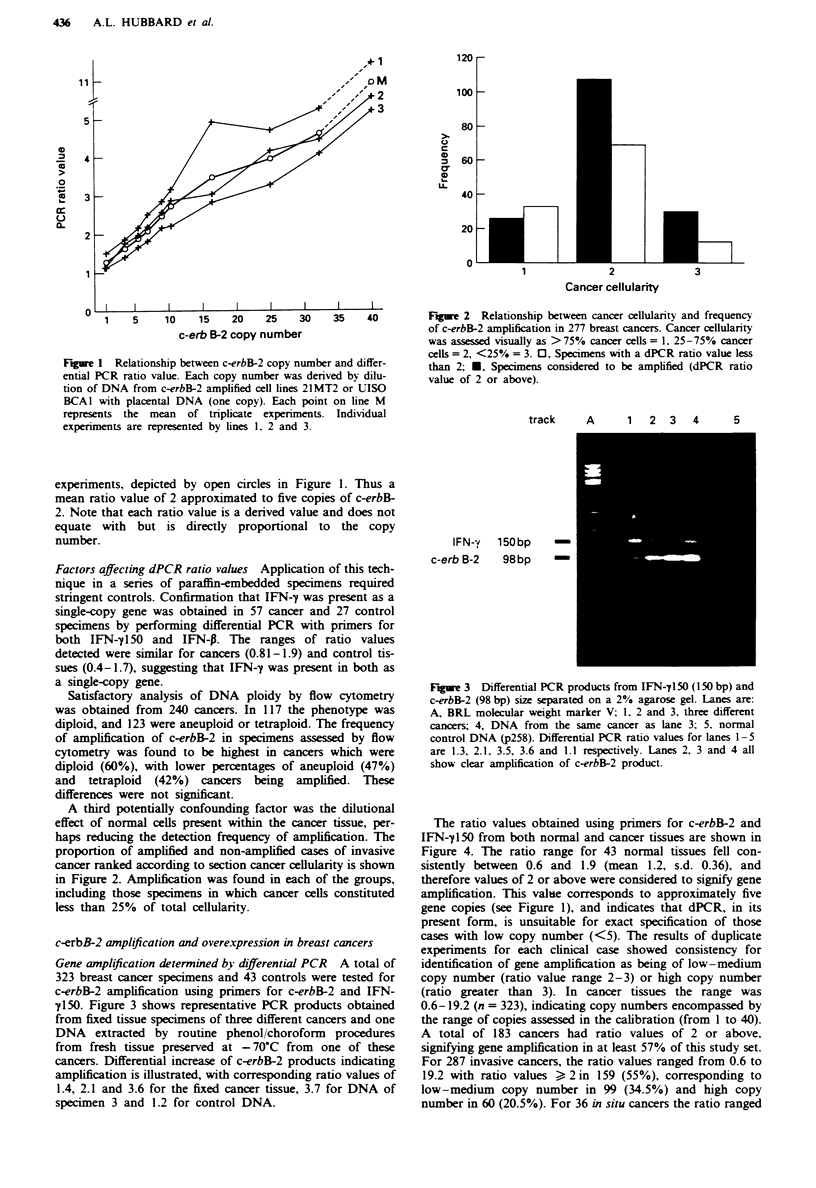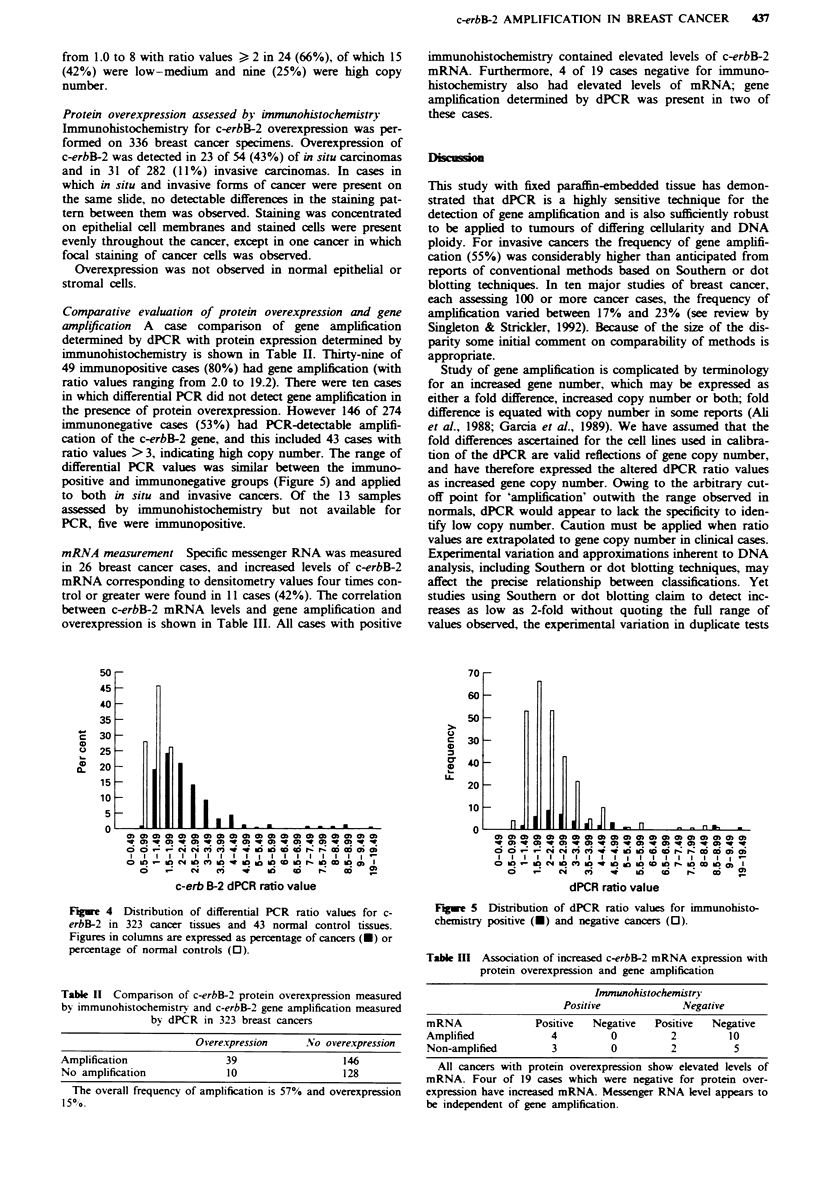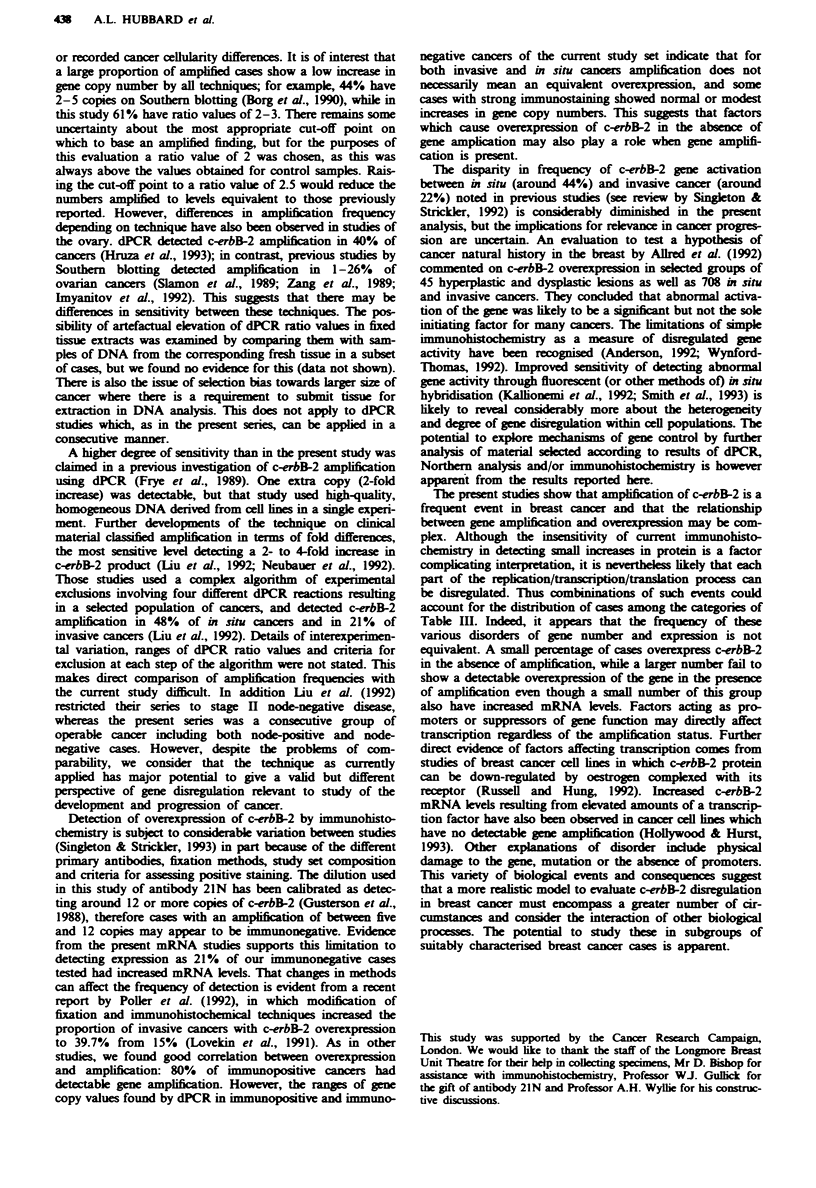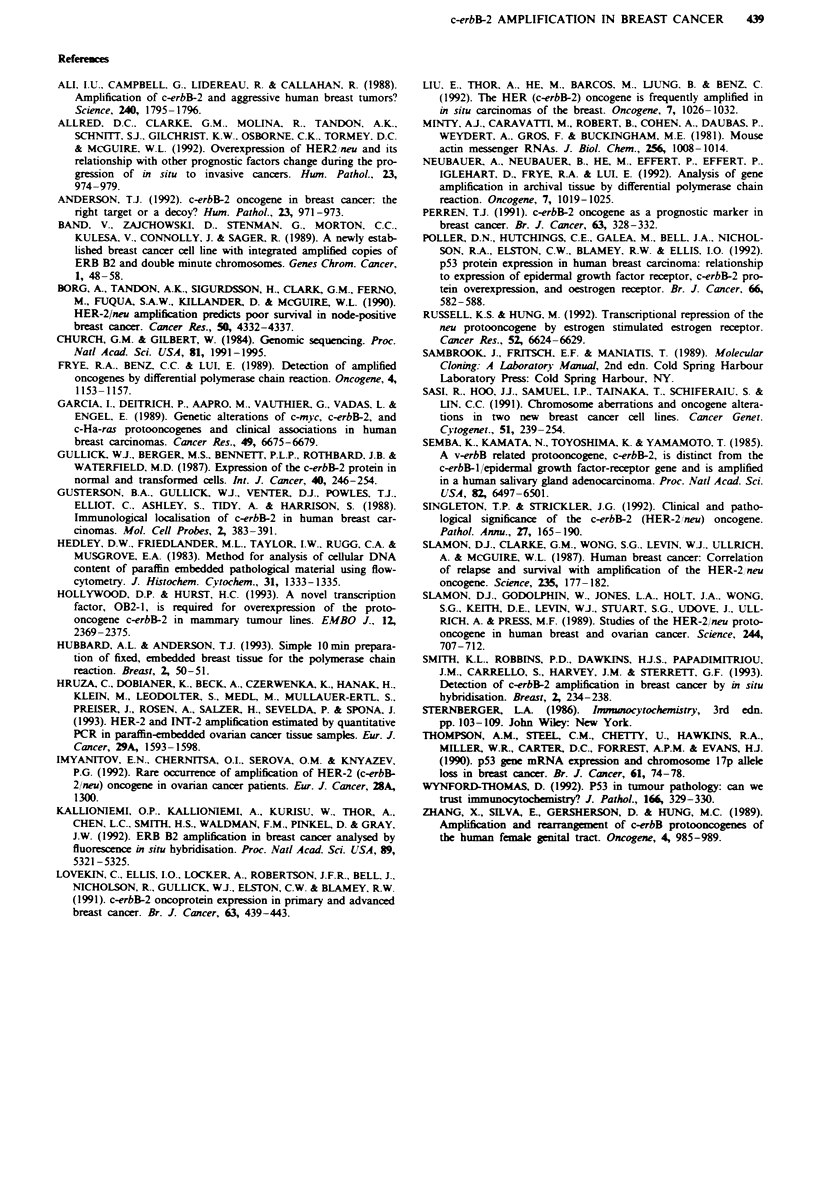# Critical determination of the frequency of c-erbB-2 amplification in breast cancer.

**DOI:** 10.1038/bjc.1994.323

**Published:** 1994-09

**Authors:** A. L. Hubbard, C. P. Doris, A. M. Thompson, U. Chetty, T. J. Anderson

**Affiliations:** Department of Pathology, University of Edinburgh, Medical School, UK.

## Abstract

**Images:**


Br. J. Cancer (1994), 70, 434–439 © Macmillan Press Ltd., 1994

### Critical determination of the frequency of c-*erb*B-2 amplification in breast cancer

A.L. Hubbard[1], C.P. Doris[1], A.M. Thompson[2], U. Chetty[3] & T.J. Anderson[1]

[1]*Department of Pathology, University of Edinburgh, Medical School, Teviot Place, Edinburgh EH8 9AG, UK;* [2]*Department of Surgery, Royal Infirmary, Edinburgh EH3 9YW, UK;* [3]*Longmore Breast Unit, Western General Hospital, Edinburgh EH4 2HU, UK.*

**Summary** Tissues from 323 methacarn-fixed and paraffin-embedded breast cancers were assessed for c-*erb*B-2 gene amplification by differential polymerase chain reaction (dPCR). The sensitivity of dPCR was ascertained using cell lines with c-*erb*B-2 amplification, and the relationship between dPCR ratio value and gene copy number was established. In clinical material the technique was not affected by the DNA contribution of normal tissue elements or by cancer DNA ploidy change. c-*erb*B-2 gene amplification was detected in 55% of invasive cancers and in 66% of *in situ* cancers. c-*erb*B-2 protein overexpression in breast cancer cells, as determined by specific immunohistochemistry, was only detected in 11% of invasive cancers and 43% of *in situ* cancers. Comparisons show that a substantial number of cancers with c-*erb*B-2 amplification lack detectable protein overexpression. This illustrates the complex nature of c-*erb*B-2 gene disregulation in cancer and suggests that multiple combinations of biological events and consequences are possible.

Disregulation of the proto-oncogene c-*erb*B-2 (also known as HER-2/*neu*) has been implicated in the aetiology of breast cancer. Since the publication of a study linking c-*erb*B-2 to poor prognosis in breast cancer patients (Slamon *et al*., 1987) there have been many studies examining c-*erb*B-2 gene amplification, mRNA production and protein overexpression. Recent reviews have collated the results from over 50 studies and found a general agreement between them on the frequency of c-*erb*B-2 disregulation in terms of gene amplification and protein overexpression, as measured by Southern blotting and immunohistochemistry respectively (Perren, 1991; Singleton & Strickler, 1992). However, there are major differences in the association of c-*erb*B-2 disregulation with histopathological features and with prognosis, making its involvement in cancer development and progression difficult to determine. It is not clear whether differences in results between studies have been the result of variations in sample selection, experimental technique or genuine biologically relevant disparity between populations.

Of the techniques for measuring gene amplification, Southern or dot blotting suffers from the disadvantages that microgram quantities of DNA are required for analysis and tissue morphology is destroyed in the extraction process. Recent advances in polymerase chain reaction (PCR) technology have made possible the analysis of minute quantities of DNA, with semiquantitative differential estimations (dPCR) demonstrating increased gene copy number in cell lines (Frye *et al*., 1989). The present work explores the sensitivity of dPCR in detecting an increased gene copy number in a large series of clinical cancers by extending the application of this technique to paraffin-embedded tissues, with a view to evaluating the relationship between c-*erb*B-2 gene amplification and expression.

### Materials and methods

### *Study set*

The study tissues (336 cases) were collected from primary operable (clinical stage I and II) breast cancers at routine operations, which included mastectomy and excisional biopsy for both palpable and non-palpable lesions. Samples were restricted to the age group 50–65 and were collected over the period of January 1988 to May 1990. They were fixed in methacarn (6:3:1 methanol–chloroform–acetic acid) overnight at 4°C, processed according to routine methods and embedded in paraffin. Control tissue (43 cases) was obtained from breast tissue distant to the lesion site or from non-cancer-bearing breasts. Pathological characterisation was taken from overall evaluation of material used for routine diagnosis, and included an evaluation of a 4 μm section immediately adjacent to sections taken for dPCR (see below). This section confirmed the nature of the tissue used in the PCR reaction, and in addition the cellularity of each specimen was assessed subjectively for the proportion of the cancer cellular content and designated as either 1 = more than 75%, 2 = 25–75% or 3 = <25%. In some cases samples of the lesion were taken and stored frozen in liquid nitrogen for RNA analysis.

### *Flow cytometric analysis*

Paraffin-embedded tumours were processed for DNA flow cytometry according to the method of Hedley *et al*. (1983). Briefly, two 50 μm sections were dewaxed using two changes of xylene and rehydrated. The tissue was incubated for 30 min at 37°C in 0.5% pepsin (Sigma) in 0.9% saline adjusted to pH 1.5 with 2 N hydrochloric acid. The isolated nuclei were counted and analysed using an EPICS C flow cytometer (Coulter Electronics, Hileah, FL, USA), after staining with 0.1% propidium iodide containing 0.004% RNAse. Ten thousand nuclei were counted at 480 nm excitation and the coefficient of variation calculated using STATPACK software (Coulter Electronics).

Ploidy was assessed as either diploid (DNA index, DI, between 0.9 and 1.10) or aneuploid (DI > 1.10 and < 1.90 or > 2.10). Tetraploids were classified as DI between 1.90 and 2.10 with more than 20% of the cells apparently in $G_2$ plus M phase of the cell cycle. For inclusion the coefficient of variation for the peak value had to be less than 8%.

### *Cell lines and culture conditions*

Human breast cancer cell lines known to have an amplification of c-*erb*B-2 were used to calibrate the relationship between differential PCR ratio values and gene copy number. The epithelial cell line 21MT2 was obtained from R. Sager (Dana-Farber Cancer Institute, Boston, MA, USA) and contains a 40-fold increase of the c-*erb*B-2 gene (Band *et al*., 1989). The cell line UISO BCA1 was obtained from R.R. Mehta (University of Illinois, Chicago, IL, USA) and contains a 10-fold increase in the c-*erb*B-2 gene (Sasi *et al*., 1991).

Correspondence: T.J. Anderson.
Received 23 November 1993; and in revised form 21 April 1994.

Each cell line was grown at 37°C in air with 5% carbon dioxide added. 21MT2 was cultured in alpha minimum essential medium (MEM) (Gibco) containing 10% fetal calf serum, 2 mM L-glutamine, 1 mM sodium pyruvate, 0.1 mM non-essential amino acids, 1 $\mu g\ ml^{-1}$ insulin, 2.8 $\mu M$ hydrocortisone 12.5 $ng\ ml^{-1}$ epidermal growth factor and 10 mM HEPES. UISO BCA1 was cultured in Glasgow's minimum essential medium (GMEM) (Gibco), 10% fetal calf serum and 2 mM L-glutamine. For calibration experiments, DNA was prepared from each cell line (Sambrook *et al.*, 1989) and was mixed with control DNA derived from normal placenta (p258, one c-*erb*-B-2 gene copy), in proportions which gave a series of known c-*erb*B-2 copy numbers. The 21MT2 DNA was diluted to give c-*erb*B-2 copy numbers of 32, 24, 16, and 8, and UISO BCA1 was diluted to give copy numbers of 9, 6, 5, 4 and 3.

*Immunohistochemistry*

Overexpression of c-*erb*B-2 was ascertained using the rabbit polyclonal antibody, 21N, to the c-*erb*B-2 protein (Gullick *et al.*, 1987). Four micron sections of fixed tissue were dried at 56°C then stained in a three-stage peroxidase–antiperoxidase technique (Sternberger, 1986). The primary antibody, 21N, was used at a concentration of 3.3 $\mu g\ ml^{-1}$ in 0.1 M Tris-buffered saline (pH 7.6) containing 5% normal swine serum. Each section was incubated at room temperature for 90 min. Endogenous peroxidase was blocked by exposure to 1% hydrogen peroxide in methanol for 30 min before staining. Overexpression of c-*erb*B-2 was defined as the presence of brown staining of surface membrane of cancer cells. To score positive, more than 10% of cells had to show moderate to strong staining. Controls included a known positive case and a negative control employing a preincubation of the antibody with its corresponding peptide (1 $mg\ ml^{-1}$).

*mRNA*

Messenger RNA was extracted from frozen tumour samples and analysed by Northern blot (Thompson *et al.*, 1990). Twenty micrograms of total RNA was denatured with formamide and formaldehyde at 55°C for 20 min and RNA species separated by electrophoresis on a 1.1% agarose gel. The RNA was transferred to a nylon filter (hybond-N, Amersham, UK) by capillary action using 10 × SSC and covalently fixed to the membrane using a UV transilluminator. To detect c-*erb*B-2 mRNA the filters were hybridised with λ107, a 1.7 kb fragment of v-*erb*B-2 (Semba *et al.*, 1985), according to the method of Church and Gilbert (1984), washed to remove non-specifically attached probe and exposed to preflashed Kodak XAR film at −70°C. Filters were stripped and reprobed with α-actin (Minty *et al.*, 1981) as an internal control for loading. The extent of hybridisation of radiolabelled probe to the mRNA species was determined using densitometry and expressed with respect to hybridisation to the actin probe.

*Primers and the polymerase chain reaction*

Primers used in the differential PCR are listed in Table I. They were DNA sequences specific for interferon gamma (IFN-γ150), c-*erb*B-2 and interferon beta (IFN-β). The single-copy reference sequence was the 150 bp sequence from the IFN-γ gene. For dPCR four 10-μm sections of fixed paraffin-embedded tissue were added to 100 μl of lysis buffer (50 mM Tris–HCl, pH 8.4, 1 mM EDTA, 0.5% Tween 20) and boiled for 8 min (Hubbard & Anderson, 1993). Differential PCR was performed on a Techne PHC3 thermal cycler incorporating 5 μl of prepared lysed paraffin section or 200 ng of extracted DNA, 0.25 μM each primer (except for primers for IFN-β, 0.125 μM), 200 mM dNTPs (Pharmacia), × 1 *Taq* polymerase buffer (Northumbria Biotechnology Limited, NBL), 1 unit of *Taq* polymerase (NBL) and 3 μCi of $[^{32}P]$CTP (New England Nuclear). Cycling parameters were one cycle of 94°C for 5 min, 50°C for 1 min, 70°C for 1 min, followed by 30 cycles of 94°C for 1 min, 50°C for 1 min, 70°C for 1 min, and one cycle of 94°C for 1 min, 50°C for 1 min, 70°C for 5 min. Duplicate PCR products were separated by size on 2% agarose gels, and stained with ethidium bromide. Visible bands were excised, finely chopped and added to 5 ml of Optiphase-safe scintillation fluid and radioactivity present assessed as counts per minute (c.p.m.) on a Beckman scintillation counter. A correction factor was applied to compensate for the differences in numbers of CTP bases between reference and test gene. All specimens were assessed in duplicate experiments.

The results from dPCR are expressed as ratio values and were calculated by averaging the c.p.m. from duplicate gel tracks and subtracting the average experimental blank. For c-*erb*B-2 a correction factor of 1.25 was applied to compensate for differences in dCTP content between IFN-γ150 (69 C bases) and c-*erb*B-2 (55 C bases). To ascertain the relative quantity of c-*erb*B-2 gene with respect to the reference gene, the corrected average c.p.m. for c-*erb*B-2 was divided by the average c.p.m. for IFN-γ150, giving in each case a result expressed as a ratio value. Similar correction factors were calculated and applied to dPCR involving amplification of IFN-β.

### Results

*Validation of differential PCR method*

*Calibration of ratio values* Differential PCR was assessed for sensitivity and reproducibility in determining gene copy number. As defined here, 'one gene copy' corresponds to the normal diploid content of one cell. The results of differential PCR on the various DNA solutions of p258 and cell lines 21MT2 and UISO BCA1 with known c-*erb*B-2 gene copy number, using primers for IFN-γ150 and c-*erb*B-2, are shown in Figure 1. A comparison of the known copy numbers in each cell line sample and differential PCR ratio values showed that increasing gene copy number resulted in increasing ratio values; mean values for 1 and 40 gene copy numbers were 1.66 and 11.46 respectively. While comparison of the ratio values obtained for given copy numbers shows some variation between experiments, there was a consistent increment in this value within each experiment. Samples with large amplifications (>32) showed increased variation between duplicate tests. For the purposes of standardisation it was considered best to work on a mean value for these

**Table I** DNA sequences of primers used in differential PCR

| *Gene* | *Sequence* | *Reference* |
|---|---|---|
| c-*erb*B-2 | | |
| Sense | 5′-CCT CTG ACG TCC ATC ATC TC-3′ | Frye *et al.* (1989) |
| Antisense | 5′-ATC TTC TGC TGC CGT CGC TT-3′ | |
| IFN-γ150 | | |
| Sense | 5′-TCT TTT CTT TCC CGA TAG GT-3′ | Frye *et al.* (1989) |
| Antisense | 5′-CTG GGA TGC TCT TCG ACC TC-3′ | |
| IFN-β | | |
| Sense | 5′-GTG TCT CCT CCA AAT TGC TC-3′ | Neubauer *et al.* (1992) |
| Antisense | 5′-GCC ACA GGA GCT TCT GAC AC-3′ | |

**Figure 1** Relationship between c-*erb*B-2 copy number and differential PCR ratio value. Each copy number was derived by dilution of DNA from c-*erb*B-2 amplified cell lines 21MT2 or UISO BCA1 with placental DNA (one copy). Each point on line M represents the mean of triplicate experiments. Individual experiments are represented by lines 1, 2 and 3.

**Figure 2** Relationship between cancer cellularity and frequency of c-*erb*B-2 amplification in 277 breast cancers. Cancer cellularity was assessed visually as >75% cancer cells = 1, 25–75% cancer cells = 2, <25% = 3. □, Specimens with a dPCR ratio value less than 2; ■, Specimens considered to be amplified (dPCR ratio value of 2 or above).

**Figure 3** Differential PCR products from IFN-γ150 (150 bp) and c-*erb*B-2 (98 bp) size separated on a 2% agarose gel. Lanes are: A, BRL molecular weight marker V; 1, 2 and 3, three different cancers; 4, DNA from the same cancer as lane 3; 5, normal control DNA (p258). Differential PCR ratio values for lanes 1–5 are 1.3, 2.1, 3.5, 3.6 and 1.1 respectively. Lanes 2, 3 and 4 all show clear amplification of c-*erb*B-2 product.

experiments, depicted by open circles in Figure 1. Thus a mean ratio value of 2 approximated to five copies of c-*erb*B-2. Note that each ratio value is a derived value and does not equate with but is directly proportional to the copy number.

*Factors affecting dPCR ratio values* Application of this technique in a series of paraffin-embedded specimens required stringent controls. Confirmation that IFN-γ was present as a single-copy gene was obtained in 57 cancer and 27 control specimens by performing differential PCR with primers for both IFN-γ150 and IFN-β. The ranges of ratio values detected were similar for cancers (0.81–1.9) and control tissues (0.4–1.7), suggesting that IFN-γ was present in both as a single-copy gene.

Satisfactory analysis of DNA ploidy by flow cytometry was obtained from 240 cancers. In 117 the phenotype was diploid, and 123 were aneuploid or tetraploid. The frequency of amplification of c-*erb*B-2 in specimens assessed by flow cytometry was found to be highest in cancers which were diploid (60%), with lower percentages of aneuploid (47%) and tetraploid (42%) cancers being amplified. These differences were not significant.

A third potentially confounding factor was the dilutional effect of normal cells present within the cancer tissue, perhaps reducing the detection frequency of amplification. The proportion of amplified and non-amplified cases of invasive cancer ranked according to section cancer cellularity is shown in Figure 2. Amplification was found in each of the groups, including those specimens in which cancer cells constituted less than 25% of total cellularity.

### *c-*erb*B-2 amplification and overexpression in breast cancers*

*Gene amplification determined by differential PCR* A total of 323 breast cancer specimens and 43 controls were tested for c-*erb*B-2 amplification using primers for c-*erb*B-2 and IFN-γ150. Figure 3 shows representative PCR products obtained from fixed tissue specimens of three different cancers and one DNA extracted by routine phenol/choroform procedures from fresh tissue preserved at −70°C from one of these cancers. Differential increase of c-*erb*B-2 products indicating amplification is illustrated, with corresponding ratio values of 1.4, 2.1 and 3.6 for the fixed cancer tissue, 3.7 for DNA of specimen 3 and 1.2 for control DNA.

The ratio values obtained using primers for c-*erb*B-2 and IFN-γ150 from both normal and cancer tissues are shown in Figure 4. The ratio range for 43 normal tissues fell consistently between 0.6 and 1.9 (mean 1.2, s.d. 0.36), and therefore values of 2 or above were considered to signify gene amplification. This value corresponds to approximately five gene copies (see Figure 1), and indicates that dPCR, in its present form, is unsuitable for exact specification of those cases with low copy number (<5). The results of duplicate experiments for each clinical case showed consistency for identification of gene amplification as being of low–medium copy number (ratio value range 2–3) or high copy number (ratio greater than 3). In cancer tissues the range was 0.6–19.2 ($n = 323$), indicating copy numbers encompassed by the range of copies assessed in the calibration (from 1 to 40). A total of 183 cancers had ratio values of 2 or above, signifying gene amplification in at least 57% of this study set. For 287 invasive cancers, the ratio values ranged from 0.6 to 19.2 with ratio values ⩾ 2 in 159 (55%), corresponding to low–medium copy number in 99 (34.5%) and high copy number in 60 (20.5%). For 36 *in situ* cancers the ratio ranged from 1.0 to 8 with ratio values $\geqslant 2$ in 24 (66%), of which 15 (42%) were low–medium and nine (25%) were high copy number.

*Protein overexpression assessed by immunohistochemistry* Immunohistochemistry for c-*erb*B-2 overexpression was performed on 336 breast cancer specimens. Overexpression of c-*erb*B-2 was detected in 23 of 54 (43%) of *in situ* carcinomas and in 31 of 282 (11%) invasive carcinomas. In cases in which *in situ* and invasive forms of cancer were present on the same slide, no detectable differences in the staining pattern between them was observed. Staining was concentrated on epithelial cell membranes and stained cells were present evenly throughout the cancer, except in one cancer in which focal staining of cancer cells was observed.

Overexpression was not observed in normal epithelial or stromal cells.

*Comparative evaluation of protein overexpression and gene amplification* A case comparison of gene amplification determined by dPCR with protein expression determined by immunohistochemistry is shown in Table II. Thirty-nine of 49 immunopositive cases (80%) had gene amplification (with ratio values ranging from 2.0 to 19.2). There were ten cases in which differential PCR did not detect gene amplification in the presence of protein overexpression. However 146 of 274 immunonegative cases (53%) had PCR-detectable amplification of the c-*erb*B-2 gene, and this included 43 cases with ratio values $>3$, indicating high copy number. The range of differential PCR values was similar between the immunopositive and immunonegative groups (Figure 5) and applied to both *in situ* and invasive cancers. Of the 13 samples assessed by immunohistochemistry but not available for PCR, five were immunopositive.

*mRNA measurement* Specific messenger RNA was measured in 26 breast cancer cases, and increased levels of c-*erb*B-2 mRNA corresponding to densitometry values four times control or greater were found in 11 cases (42%). The correlation between c-*erb*B-2 mRNA levels and gene amplification and overexpression is shown in Table III. All cases with positive immunohistochemistry contained elevated levels of c-*erb*B-2 mRNA. Furthermore, 4 of 19 cases negative for immunohistochemistry also had elevated levels of mRNA; gene amplification determined by dPCR was present in two of these cases.

### Discussion

This study with fixed paraffin-embedded tissue has demonstrated that dPCR is a highly sensitive technique for the detection of gene amplification and is also sufficiently robust to be applied to tumours of differing cellularity and DNA ploidy. For invasive cancers the frequency of gene amplification (55%) was considerably higher than anticipated from reports of conventional methods based on Southern or dot blotting techniques. In ten major studies of breast cancer, each assessing 100 or more cancer cases, the frequency of amplification varied between 17% and 23% (see review by Singleton & Strickler, 1992). Because of the size of the disparity some initial comment on comparability of methods is appropriate.

Study of gene amplification is complicated by terminology for an increased gene number, which may be expressed as either a fold difference, increased copy number or both; fold difference is equated with copy number in some reports (Ali *et al*., 1988; Garcia *et al*., 1989). We have assumed that the fold differences ascertained for the cell lines used in calibration of the dPCR are valid reflections of gene copy number, and have therefore expressed the altered dPCR ratio values as increased gene copy number. Owing to the arbitrary cut-off point for 'amplification' outwith the range observed in normals, dPCR would appear to lack the specificity to identify low copy number. Caution must be applied when ratio values are extrapolated to gene copy number in clinical cases. Experimental variation and approximations inherent to DNA analysis, including Southern or dot blotting techniques, may affect the precise relationship between classifications. Yet studies using Southern or dot blotting claim to detect increases as low as 2-fold without quoting the full range of values observed, the experimental variation in duplicate tests

**Figure 4** Distribution of differential PCR ratio values for c-*erb*B-2 in 323 cancer tissues and 43 normal control tissues. Figures in columns are expressed as percentage of cancers (■) or percentage of normal controls (□).

**Figure 5** Distribution of dPCR ratio values for immunohistochemistry positive (■) and negative cancers (□).

**Table II** Comparison of c-*erb*B-2 protein overexpression measured by immunohistochemistry and c-*erb*B-2 gene amplification measured by dPCR in 323 breast cancers

| | *Overexpression* | *No overexpression* |
|---|---|---|
| Amplification | 39 | 146 |
| No amplification | 10 | 128 |

The overall frequency of amplification is 57% and overexpression 15%.

**Table III** Association of increased c-*erb*B-2 mRNA expression with protein overexpression and gene amplification

| | *Immunohistochemistry* | | | |
|---|---|---|---|---|
| | *Positive* | | *Negative* | |
| mRNA | Positive | Negative | Positive | Negative |
| Amplified | 4 | 0 | 2 | 10 |
| Non-amplified | 3 | 0 | 2 | 5 |

All cancers with protein overexpression show elevated levels of mRNA. Four of 19 cases which were negative for protein overexpression have increased mRNA. Messenger RNA level appears to be independent of gene amplification.

or recorded cancer cellularity differences. It is of interest that a large proportion of amplified cases show a low increase in gene copy number by all techniques; for example, 44% have 2–5 copies on Southern blotting (Borg *et al.*, 1990), while in this study 61% have ratio values of 2–3. There remains some uncertainty about the most appropriate cut-off point on which to base an amplified finding, but for the purposes of this evaluation a ratio value of 2 was chosen, as this was always above the values obtained for control samples. Raising the cut-off point to a ratio value of 2.5 would reduce the numbers amplified to levels equivalent to those previously reported. However, differences in amplification frequency depending on technique have also been observed in studies of the ovary. dPCR detected c-*erb*B-2 amplification in 40% of cancers (Hruza *et al.*, 1993); in contrast, previous studies by Southern blotting detected amplification in 1–26% of ovarian cancers (Slamon *et al.*, 1989; Zang *et al.*, 1989; Imyanitov *et al.*, 1992). This suggests that there may be differences in sensitivity between these techniques. The possibility of artefactual elevation of dPCR ratio values in fixed tissue extracts was examined by comparing them with samples of DNA from the corresponding fresh tissue in a subset of cases, but we found no evidence for this (data not shown). There is also the issue of selection bias towards larger size of cancer where there is a requirement to submit tissue for extraction in DNA analysis. This does not apply to dPCR studies which, as in the present series, can be applied in a consecutive manner.

A higher degree of sensitivity than in the present study was claimed in a previous investigation of c-*erb*B-2 amplification using dPCR (Frye *et al.*, 1989). One extra copy (2-fold increase) was detectable, but that study used high-quality, homogeneous DNA derived from cell lines in a single experiment. Further developments of the technique on clinical material classified amplification in terms of fold differences, the most sensitive level detecting a 2- to 4-fold increase in c-*erb*B-2 product (Liu *et al.*, 1992; Neubauer *et al.*, 1992). Those studies used a complex algorithm of experimental exclusions involving four different dPCR reactions resulting in a selected population of cancers, and detected c-*erb*B-2 amplification in 48% of *in situ* cancers and in 21% of invasive cancers (Liu *et al.*, 1992). Details of interexperimental variation, ranges of dPCR ratio values and criteria for exclusion at each step of the algorithm were not stated. This makes direct comparison of amplification frequencies with the current study difficult. In addition Liu *et al.* (1992) restricted their series to stage II node-negative disease, whereas the present series was a consecutive group of operable cancer including both node-positive and node-negative cases. However, despite the problems of comparability, we consider that the technique as currently applied has major potential to give a valid but different perspective of gene disregulation relevant to study of the development and progression of cancer.

Detection of overexpression of c-*erb*B-2 by immunohistochemistry is subject to considerable variation between studies (Singleton & Strickler, 1993) in part because of the different primary antibodies, fixation methods, study set composition and criteria for assessing positive staining. The dilution used in this study of antibody 21N has been calibrated as detecting around 12 or more copies of c-*erb*B-2 (Gusterson *et al.*, 1988), therefore cases with an amplification of between five and 12 copies may appear to be immunonegative. Evidence from the present mRNA studies supports this limitation to detecting expression as 21% of our immunonegative cases tested had increased mRNA levels. That changes in methods can affect the frequency of detection is evident from a recent report by Poller *et al.* (1992), in which modification of fixation and immunohistochemical techniques increased the proportion of invasive cancers with c-*erb*B-2 overexpression to 39.7% from 15% (Lovekin *et al.*, 1991). As in other studies, we found good correlation between overexpression and amplification: 80% of immunopositive cancers had detectable gene amplification. However, the ranges of gene copy values found by dPCR in immunopositive and immunonegative cancers of the current study set indicate that for both invasive and *in situ* cancers amplification does not necessarily mean an equivalent overexpression, and some cases with strong immunostaining showed normal or modest increases in gene copy numbers. This suggests that factors which cause overexpression of c-*erb*B-2 in the absence of gene amplication may also play a role when gene amplification is present.

The disparity in frequency of c-*erb*B-2 gene activation between *in situ* (around 44%) and invasive cancer (around 22%) noted in previous studies (see review by Singleton & Strickler, 1992) is considerably diminished in the present analysis, but the implications for relevance in cancer progression are uncertain. An evaluation to test a hypothesis of cancer natural history in the breast by Allred *et al.* (1992) commented on c-*erb*B-2 overexpression in selected groups of 45 hyperplastic and dysplastic lesions as well as 708 *in situ* and invasive cancers. They concluded that abnormal activation of the gene was likely to be a significant but not the sole initiating factor for many cancers. The limitations of simple immunohistochemistry as a measure of disregulated gene activity have been recognised (Anderson, 1992; Wynford-Thomas, 1992). Improved sensitivity of detecting abnormal gene activity through fluorescent (or other methods of) *in situ* hybridisation (Kallionemi *et al.*, 1992; Smith *et al.*, 1993) is likely to reveal considerably more about the heterogeneity and degree of gene disregulation within cell populations. The potential to explore mechanisms of gene control by further analysis of material selected according to results of dPCR, Northern analysis and/or immunohistochemistry is however apparent from the results reported here.

The present studies show that amplification of c-*erb*B-2 is a frequent event in breast cancer and that the relationship between gene amplification and overexpression may be complex. Although the insensitivity of current immunohistochemistry in detecting small increases in protein is a factor complicating interpretation, it is nevertheless likely that each part of the replication/transcription/translation process can be disregulated. Thus combininations of such events could account for the distribution of cases among the categories of Table III. Indeed, it appears that the frequency of these various disorders of gene number and expression is not equivalent. A small percentage of cases overexpress c-*erb*B-2 in the absence of amplification, while a larger number fail to show a detectable overexpression of the gene in the presence of amplification even though a small number of this group also have increased mRNA levels. Factors acting as promoters or suppressors of gene function may directly affect transcription regardless of the amplification status. Further direct evidence of factors affecting transcription comes from studies of breast cancer cell lines in which c-*erb*B-2 protein can be down-regulated by oestrogen complexed with its receptor (Russell and Hung, 1992). Increased c-*erb*B-2 mRNA levels resulting from elevated amounts of a transcription factor have also been observed in cancer cell lines which have no detectable gene amplification (Hollywood & Hurst, 1993). Other explanations of disorder include physical damage to the gene, mutation or the absence of promoters. This variety of biological events and consequences suggest that a more realistic model to evaluate c-*erb*B-2 disregulation in breast cancer must encompass a greater number of circumstances and consider the interaction of other biological processes. The potential to study these in subgroups of suitably characterised breast cancer cases is apparent.

This study was supported by the Cancer Research Campaign, London. We would like to thank the staff of the Longmore Breast Unit Theatre for their help in collecting specimens, Mr D. Bishop for assistance with immunohistochemistry, Professor W.J. Gullick for the gift of antibody 21N and Professor A.H. Wyllie for his constructive discussions.

**References**

ALI, I.U., CAMPBELL, G., LIDEREAU, R. & CALLAHAN, R. (1988). Amplification of c-*erb*B-2 and aggressive human breast tumors? *Science*, **240**, 1795-1796.

ALLRED, D.C., CLARKE, G.M., MOLINA, R., TANDON, A.K., SCHNITT, S.J., GILCHRIST, K.W., OSBORNE, C.K., TORMEY, D.C. & MCGUIRE, W.L. (1992). Overexpression of HER2/*neu* and its relationship with other prognostic factors change during the progression of *in situ* to invasive cancers. *Hum. Pathol.*, **23**, 974-979.

ANDERSON, T.J. (1992). c-*erb*B-2 oncogene in breast cancer: the right target or a decoy? *Hum. Pathol.*, **23**, 971-973.

BAND, V., ZAJCHOWSKI, D., STENMAN, G., MORTON, C.C., KULESA, V., CONNOLLY, J. & SAGER, R. (1989). A newly established breast cancer cell line with integrated amplified copies of ERB B2 and double minute chromosomes. *Genes Chrom. Cancer*, **1**, 48-58.

BORG, A., TANDON, A.K., SIGURDSSON, H., CLARK, G.M., FERNO, M., FUQUA, S.A.W., KILLANDER, D. & MCGUIRE, W.L. (1990). HER-2/*neu* amplification predicts poor survival in node-positive breast cancer. *Cancer Res.*, **50**, 4332-4337.

CHURCH, G.M. & GILBERT, W. (1984). Genomic sequencing. *Proc. Natl Acad. Sci. USA*, **81**, 1991-1995.

FRYE, R.A., BENZ, C.C. & LUI, E. (1989). Detection of amplified oncogenes by differential polymerase chain reaction. *Oncogene*, **4**, 1153-1157.

GARCIA, I., DEITRICH, P., AAPRO, M., VAUTHIER, G., VADAS, L. & ENGEL, E. (1989). Genetic alterations of c-*myc*, c-*erb*B-2, and c-Ha-*ras* protooncogenes and clinical associations in human breast carcinomas. *Cancer Res.*, **49**, 6675-6679.

GULLICK, W.J., BERGER, M.S., BENNETT, P.L.P., ROTHBARD, J.B. & WATERFIELD, M.D. (1987). Expression of the c-*erb*B-2 protein in normal and transformed cells. *Int. J. Cancer*, **40**, 246-254.

GUSTERSON, B.A., GULLICK, W.J., VENTER, D.J., POWLES, T.J., ELLIOT, C., ASHLEY, S., TIDY, A. & HARRISON, S. (1988). Immunological localisation of c-*erb*B-2 in human breast carcinomas. *Mol. Cell Probes*, **2**, 383-391.

HEDLEY, D.W., FRIEDLANDER, M.L., TAYLOR, I.W., RUGG, C.A. & MUSGROVE, E.A. (1983). Method for analysis of cellular DNA content of paraffin embedded pathological material using flow-cytometry. *J. Histochem. Cytochem.*, **31**, 1333-1335.

HOLLYWOOD, D.P. & HURST, H.C. (1993). A novel transcription factor, OB2-1, is required for overexpression of the proto-oncogene c-*erb*B-2 in mammary tumour lines. *EMBO J.*, **12**, 2369-2375.

HUBBARD, A.L. & ANDERSON, T.J. (1993). Simple 10 min preparation of fixed, embedded breast tissue for the polymerase chain reaction. *Breast*, **2**, 50-51.

HRUZA, C., DOBIANER, K., BECK, A., CZERWENKA, K., HANAK, H., KLEIN, M., LEODOLTER, S., MEDL, M., MULLAUER-ERTL, S., PREISER, J., ROSEN, A., SALZER, H., SEVELDA, P. & SPONA, J. (1993). HER-2 and INT-2 amplification estimated by quantitative PCR in paraffin-embedded ovarian cancer tissue samples. *Eur. J. Cancer*, **29A**, 1593-1598.

IMYANITOV, E.N., CHERNITSA, O.I., SEROVA, O.M. & KNYAZEV, P.G. (1992). Rare occurrence of amplification of HER-2 (c-*erb*B-2/*neu*) oncogene in ovarian cancer patients. *Eur. J. Cancer*, **28A**, 1300.

KALLIONIEMI, O.P., KALLIONIEMI, A., KURISU, W., THOR, A., CHEN, L.C., SMITH, H.S., WALDMAN, F.M., PINKEL, D. & GRAY, J.W. (1992). ERB B2 amplification in breast cancer analysed by fluorescence *in situ* hybridisation. *Proc. Natl Acad. Sci. USA*, **89**, 5321-5325.

LOVEKIN, C., ELLIS, I.O., LOCKER, A., ROBERTSON, J.F.R., BELL, J., NICHOLSON, R., GULLICK, W.J., ELSTON, C.W. & BLAMEY, R.W. (1991). c-*erb*B-2 oncoprotein expression in primary and advanced breast cancer. *Br. J. Cancer*, **63**, 439-443.

LIU, E., THOR, A., HE, M., BARCOS, M., LJUNG, B. & BENZ, C. (1992). The HER (c-*erb*B-2) oncogene is frequently amplified in *in situ* carcinomas of the breast. *Oncogene*, **7**, 1026-1032.

MINTY, A.J., CARAVATTI, M., ROBERT, B., COHEN, A., DAUBAS, P., WEYDERT, A., GROS, F. & BUCKINGHAM, M.E. (1981). Mouse actin messenger RNAs. *J. Biol. Chem.*, **256**, 1008-1014.

NEUBAUER, A., NEUBAUER, B., HE, M., EFFERT, P., EFFERT, P., IGLEHART, D., FRYE, R.A. & LUI, E. (1992). Analysis of gene amplification in archival tissue by differential polymerase chain reaction. *Oncogene*, **7**, 1019-1025.

PERREN, T.J. (1991). c-*erb*B-2 oncogene as a prognostic marker in breast cancer. *Br. J. Cancer*, **63**, 328-332.

POLLER, D.N., HUTCHINGS, C.E., GALEA, M., BELL, J.A., NICHOLSON, R.A., ELSTON, C.W., BLAMEY, R.W. & ELLIS, I.O. (1992). p53 protein expression in human breast carcinoma: relationship to expression of epidermal growth factor receptor, c-*erb*B-2 protein overexpression, and oestrogen receptor. *Br. J. Cancer*, **66**, 582-588.

RUSSELL, K.S. & HUNG, M. (1992). Transcriptional repression of the *neu* protooncogene by estrogen stimulated estrogen receptor. *Cancer Res.*, **52**, 6624-6629.

SAMBROOK, J., FRITSCH, E.F. & MANIATIS, T. (1989). *Molecular Cloning: A Laboratory Manual*, 2nd edn. Cold Spring Harbour Laboratory Press: Cold Spring Harbour, NY.

SASI, R., HOO, J.J., SAMUEL, I.P., TAINAKA, T., SCHIFERAIU, S. & LIN, C.C. (1991). Chromosome aberrations and oncogene alterations in two new breast cancer cell lines. *Cancer Genet. Cytogenet.*, **51**, 239-254.

SEMBA, K., KAMATA, N., TOYOSHIMA, K. & YAMAMOTO, T. (1985). A v-*erb*B related protooncogene, c-*erb*B-2, is distinct from the c-*erb*B-1/epidermal growth factor-receptor gene and is amplified in a human salivary gland adenocarcinoma. *Proc. Natl Acad. Sci. USA*, **82**, 6497-6501.

SINGLETON, T.P. & STRICKLER, J.G. (1992). Clinical and pathological significance of the c-*erb*B-2 (HER-2/*neu*) oncogene. *Pathol. Annu.*, **27**, 165-190.

SLAMON, D.J., CLARKE, G.M., WONG, S.G., LEVIN, W.J., ULLRICH, A. & MCGUIRE, W.L. (1987). Human breast cancer: Correlation of relapse and survival with amplification of the HER-2/*neu* oncogene. *Science*, **235**, 177-182.

SLAMON, D.J., GODOLPHIN, W., JONES, L.A., HOLT, J.A., WONG, S.G., KEITH, D.E., LEVIN, W.J., STUART, S.G., UDOVE, J., ULLRICH, A. & PRESS, M.F. (1989). Studies of the HER-2/*neu* proto-oncogene in human breast and ovarian cancer. *Science*, **244**, 707-712.

SMITH, K.L., ROBBINS, P.D., DAWKINS, H.J.S., PAPADIMITRIOU, J.M., CARRELLO, S., HARVEY, J.M. & STERRETT, G.F. (1993). Detection of c-*erb*B-2 amplification in breast cancer by *in situ* hybridisation. *Breast*, **2**, 234-238.

STERNBERGER, L.A. (1986). *Immunocytochemistry*, 3rd edn. pp. 103-109. John Wiley: New York.

THOMPSON, A.M., STEEL, C.M., CHETTY, U., HAWKINS, R.A., MILLER, W.R., CARTER, D.C., FORREST, A.P.M. & EVANS, H.J. (1990). p53 gene mRNA expression and chromosome 17p allele loss in breast cancer. *Br. J. Cancer*, **61**, 74-78.

WYNFORD-THOMAS, D. (1992). P53 in tumour pathology: can we trust immunocytochemistry? *J. Pathol.*, **166**, 329-330.

ZHANG, X., SILVA, E., GERSHERSON, D. & HUNG, M.C. (1989). Amplification and rearrangement of c-*erb*B protooncogenes of the human female genital tract. *Oncogene*, **4**, 985-989.